# Supplementary material for: Enhanced Case Detection and Improved Diagnosis of PKDL in a Kala-azar-Endemic Area of Bangladesh
Source: PLoS Negl Trop Dis. 2010 Oct 5;4(10):e832. doi: 10.1371/journal.pntd.0000832 (PMC2950134; doi:10.1371/journal.pntd.0000832)
Supplement: Checklist S1 — STROBE Checklist (0.23 MB DOC) [file pntd.0000832.s001.doc]

**Title Page**

Enhanced case detection and improved diagnosis of PKDL in a kala-azar-endemic area of Bangladesh

Dinesh Mondal^1^, Kamrul Nahar Nasrin^2^, M. Mamun Huda^1^, Mamun Kabir^1^, Md. Shakhawat Hossain^1^, Axel Kroeger ^3, 4^, Tania Thomas^5^, and Rashidul Haque^1^

^1^Parasitology Laboratory, Laboratory Sciences Division, ICDDR,B, Mohakhali, Dhaka1212, Bangladesh

^2^Department of Microbiology, Bangabandhu Sheikh Mujib Medical University , Dhaka, Bangladesh

^3^Special Programme for Research and Training in Tropical Diseases , World Health Organization, Geneva, Switzerland

^4^Disease Control Strategy Group, Liverpool School of Tropical Medicine, Pembroke Place, Liverpool, UK

^5^Division of Infectious Diseases and International Health, University of Virginia, Charlottesville, VA, USA

Corresponding author:

Dr. Dinesh Mondal

Associate Scientist, Parasitology Laboratory, Laboratory Sciences Division, International Centre for Diarrhoeal Disease Research, Bangladesh Mohakhali, Dhaka1212, Bangladesh,

Phone: +880-2-88160523 ext 2417

Fax: +880-2-8812529

E-mail: [din63d@icddrb.org](mailto:din63d@icddrb.org)

**Abstract**

**Objectives:** To support the Bangladesh National Kala-azar Elimination Programme (NKEP), we investigated the feasibility of using of trained village volunteers for detecting post-kala-azar dermal leishmaniasis (PKDL) cases , using of polymerase chain reaction (PCR) for confirmation of diagnosis and treatment compliance by PKDL patients in Kanthal union of Trishal sub-district, Mymensingh, Bangladesh.

**Methods**: In this cross-sectional study, Field Research Assistants (FRAs) conducted census in the study area, and the research team trained village volunteers on how to look for PKDL suspects. The trained village volunteers (TVVs)visited each household in the study area for PKDL suspects and referred the suspected PKDL cases to the study clinic. The suspected cases underwent physical examinations by a qualified doctor and rK39 strip testing by the FRAs, and if positive, slit skin examination (SSE), culture, and PCR of skin specimens and peripheral buffy coat were done. Those with evidence of *Leishmania donovani* (LD) were referred for treatment. All the cases were followed for one year.

**Results:** The total population of the study area was 29,226 from 6,566 households. The TVVs referred 52 PKDL suspects. Probable PKDL was diagnosed in 18 of the 52 PKDL suspect cases, and PKDL was confirmed in 9 of the 18 probable PKDL cases. The prevalence of probable PKDL was 6.2 per 10,000 people in the study area. Thirteen PKDL suspects self-reported from outside the study area, and probable and confirmed PKDL was diagnosed in 10 of the 13 suspects and in 5 of 10 probable PKDL cases respectively. All probable PKDL cases had hypopigmented macules. The median time for PKDL development was 36 months ( IQR, 24-48) . Evidence of the LD parasite was documented by SSE and PCR in 3.6% and 64.3% of the cases respectively. PCR positivity was associated with gender and severity of disease. Those who were untreated had an increased risk ( odds ratio=3.33, 95%CI 1.29-8.59) of having persistent skin lesions compared to those who were treated. Patients’ treatment-seeking behavior and treatment compliance were poor.

**Conclusion:** Improved detection of PKDL cases by TVVs is feasible and useful. The NKEP should promote PCR for the diagnosis of PKDL and should find ways for improving treatment compliance by patients.

Key words: Post-kala-azar dermal leishmaniasis; Kala-azar; Bangladesh; Case detection; PCR; Treatment compliance

**Author summary**

PKDL is a skin disorder which usually develops in 10-20% and about 60% of patients with visceral leishmaniasis (VL) after treatment respectively in the Indian sub-continent and Sudan. However, cases among people without prior VL have also been reported. Except skin lesion, PKDL patients are healthy and usually do not feel sick. However, persistence of a few PKDL cases is sufficient to initiate a new epidemic of anthroponotic VL. Thus, identifying and treating people with PKDL are a key strategy for the elimination of kala-azar. Diagnosis of PKDL relies upon clinical criteria and a serological test which is not specific for PKDL. The use of the existing laboratory diagnostic tools for confirmation of PKDL among PKDL suspects is unknown. In the Indian subcontinent, PKDL is not self-limited and needs to be treated with sodium stibogluconate injections for 4-6 months. No data are available relating to treatment compliance by patients, particularly in Bangladesh. The results of the present study showed that trained village volunteers were useful for identifying PKDL suspects, and diagnostic confirmation improved with the use of PCR. However, patients’ adherence to prescribed treatment was poor.

**Introduction**

Post- kala-azar dermal leishmanisis (PKDL), is a skin disorder, usually develops in 10-20% and about 60% of patients with visceral leishmaniasis (VL)/kala-azar after treatment, respectively, in the Indian sub-continent and Sudan [1]. It has also been reported in individuals without prior history of VL and those undergoing treatment for VL [1-6]. The protozoan parasite *Leishmania donovani* (LD) is the only causative agent. Clinical manifestations of PKDL are macular, maculo-papular, and nodular rash in people who are otherwise well [1] and may be confused with leprosy. Since PKDL is the only interepidemic reservoir of anthroponotic VL, the existence of a few cases is sufficient to trigger a new epidemic of VL in a given community [1,4,7]. Thus, identification and treatment of PKDL is an essential strategy in eliminating VL.

In 2005, Health Ministers of Bangladesh, India, and Nepal signed a Memorandum of Understanding for the elimination of VL from the Indian sub-continent by 2015 [8]. Active VL and PKDL case detection and their proper management are two important strategies of the elimination program [8]. Until now, no definite method has been identified for active VL and PKDL case detection but one proposed plan includes a house-to-house search for cases by public-health workers. Such a method is expensive and requires alternative strategies for VL and PKDL active case detection [9].

In the Indian sub-continent, PKDL was first described by Brahmchari in 1922 [10]. Since then, nearly 90 years have passed and no gold standard diagnostic method could be developed for PKDL; diagnosis, thus, relies on clinical criteria [1-6]. PCR of slit skin scraping specimens has demonstrated high sensitivity for diagnosing PKDL in a laboratory-based study [11]. However, its use in clinically-diagnosed PKDL patients is unknown. PCR testing of peripheral blood buffy coat has been found to be a highly-sensitive method for the diagnosis of VL [12,13], and theoretically, it may help also confirm PKDL.

In Bangladesh, in the post-malaria-eradication era, the first reports on PKDL were from hospital- based studies [14,15]. Until now there has been limited information on the burden of PKDL in the VL-endemic communities of Bangladesh [4]. Preliminary results of an ongoing surveillance study of PKDL in Fulbaria, Mymensingh, Bangladesh, showed that the burden of PKDL was high and presents a challenge for the National Kala-azar Elimination Programme (NKEP) [4]. More information on the burden of PKDL will help the NKEP to develop adequate national strategies for controlling PKDL.

We, therefore, studied the feasibility of using trained village volunteers (TVVs) for detecting suspected PKDL cases; estimated the prevalence of PKDL in Kanthal Union, Trishal, a VL-endemic area of Bangladesh; described the clinical features of these patients; evaluated the contribution of PCR for the confirmation of PKDL diagnosis in clinically-diagnosed PKDL cases ; and investigated the patients’ compliance to treatment. .

**Methods**

***Study area and population***

We conducted a study in Kanthal union, Trishal sub-district under Mymensingh district, Bangladesh. During January-March 2008, trained field research assistants performed a census in Kanthal union. During the census, field research assistants assessed household number and the number of people in each household. They also asked and recorded whether any member within the family suffered from kala-azar in the past and if any of them currently had skin rash.

***Trained village volunteers***

After consulting the local community leaders and obtaining their consent, one community volunteer was selected from each ward. The research team trained the volunteers on what was PKDL, how did a PKDL case looks like, and how to look for the suspected PKDL case ( see definition below). A two-day training was imported to the volunteers, and pictures of skin lesions from PKDL patients from published literature and textbooks were used. During April-May 2008, nine TVVs visited each household at least once searching for suspected PKDL cases and, if found, referred the case to the study clinic. Household members who had a history of VL but were not present during the home-visits were invited to visit to the study clinic for assessment. Additionally, the study physician assessed patients with a history of VL and skin rash who lived in villages outside the study area. Most of these patients were directed to our study clinic from nearby public union health posts where rK39 tests were not available.

***Evaluation and procedures***

The study physician examined the suspected PKDL cases and requested an rK39 strip test (Kala-azar Detect ^TM^ Rapid Test, InBios International, Seattle, WA, USA) as needed. A trained field research assistant performed the rK39 strip test as per the manufacturer’s instructions. If the results were positive, the patients were considered probable PKDL cases (see definition below) and were requested to undergo slit skin scraping and blood collection. A physician-microbiologist from the Bangabandhu Sheikh Mujib Medical University (BSMMU) in Dhaka performed slit skin scraping and collected skin specimens for staining, culture, and PCR test in the study clinic. The study physician collected up to 5 mL of venous blood in EDTA tubes and gently shook. After collection, the blood and skin specimens were transported to the Parasitology Laboratory of the International Centre for Diarrhoeal Disease Research, Bangladesh (ICDDR,B) within 3-4 hours maintaining cold chain. PCR tests were performed in the Parasitology Laboratory of ICDDR,B on the following day, and the study physician was informed about PCR results immediately after testing.

***Treatment and follow-up***

We referred all cases positive for the LD parasite or positive for LD DNA by PCR to the Upazila Health Complex for treatment as per the national guideline for kala-azar elimination [17]. After referral, we followed up patients to find out whether they went to the hospital for admission, and if admitted, whether they completed full treatment courses. Further, we followed all the probable PKDL patients after one year from the date of their referral by household-visit and collected information on the status of their skin rash. Patients who were rK39 test-negative were referred to the Mymensingh Medical College Hospital for further medical consultation.

***Laboratory methods***

**Collection of slit skin scraping for staining, culture, and PCR**

The affected area of the skin was cleaned with 70% v/v alcohol and allowed to dry completely. The edge of the lesion was squeezed firmly between the finger and the thumb to drain the area of blood. Using a sterile scalpel blade, a small incision was made into the dermis; any blood was blotted away. The cut surface was then scraped in an outward direction to obtain the tissue fluid and cells for the following procedures:

1. Preparation of slides and process of staining
   The materials were thinly spread on three-clean glass slides using a circular motion working outwards to avoid damaging any parasites. When the smears dried, two slides were fixed with a few drops of absolute methanol for 2-3 minutes. The remaining slide was heat- fixed by holding it, smear side up, over the flame of a spirit lamp for a few seconds. After fixation, the slides were stored and transported to the laboratory of BSMMU and stained with modified Ziehl-Neelsen (Z-N) staining (using 5% sulphuric acid, H_2_SO_4_). It was then examined under oil immersion lens using 100x oil immersion objectives to look for *Mycobacterium leprae*. The methanol-fixed slides were stained with Giemsa stain and examined for LD bodies.
2. Collection of slit skin scrapings for PCR

Collection of slit skin scraping specimens for PCR was done as described previously [16], with little modification which included collection of skin scrapings using a sterile cotton swab to maximize the collection of tissue fluid and cells. After cutting the edge of the lesion, a sterile cotton swab was used for absorbing the tissue fluid and placed into an appendorf tube containing 500 µL of NET buffer (150 Mm Nacl, 15 Mm Tris-HCl [pH-8.30]). The cotton swab was kept in the tube for one hour before removal; and the buffer solution was preserved at -20 °C at the Parasitology Laboratory of ICDDR,B until PCR was performed .

1. Collection of slit skin scrapings for culture

Slit skin scrapings were collected with sterile stainless steel wire loops and inoculated in the Novy-MacNeal-Nicolle medium which was transported to the Microbiology Laboratory of BSMMU, in a air-conditioned car maintaining the temperature at 22 °C and was incubated at 22–24 °C in the laboratory. Culture was examined weekly and was kept up to four weeks before discarding as negative.

**Preparation of buffy coat**

After arrival at the laboratory, blood samples were centrifuged at 8000 rpm for 10 minutes at room temperature, and 500 μL of buffy coat was collected from the middle layer of the tube containing concentrated leukocytes. The buffy coat was kept in a 1.5-mL sterile microcentrifuge tube and preserved at -20 ºC for DNA extraction and PCR amplification.

**DNA extraction from buffy coat and skin slit**

Buffy coat DNA was extracted for PCR using QIAamp DNA blood mini kit (Qiagen, Hilden, Germany, Cat. no. 51106) as per the manufacturer’s instructions. The DNA was eluted in 0.2 mL of AE buffer (supplied with the Qiagen kit). DNA was extracted from the skin slit also following the manufacture’s instructions, except that the proteinase K digestion was carried out for 60 minutes at 50 °C. The DNA was eluted in 0.2 mL of AE buffer (supplied with the Qiagen kit). The purity of the DNA was satisfactory since a ratio of OD at A260/A280 was within 1.7-1.9 for all DNA samples. We used molecular-grade water instead of blood as an extraction control for checking for carry-over contamination in every run of DNA extraction and PCR amplification.

**PCR methods**

*Leishmania-*specific nested PCR (Ln-PCR) was performed to detect LD DNA in peripheral buffy coat and skin specimens using 2 μL of extracted DNA by the method described previously [13], with primers targeting the parasite’s SSU-rRNA region [12]. For the first PCR run, we used Kinetoplastida-specific primers (R221 5′-GGTTCCTTTCCTGATTTACG-3′ and R332 5′- GGCCGGTAAAGGCCGAATAG-3′ ). For the second PCR, 1 μL of 1:50 dilution of the first PCR product was used as a template in the presence of 0.15 μmol/L of the *Leishmania-*specific primers R223 and R333.

**Microscopy**

**Microscopy for LD bodies:** At least two experts examined the Giemsa stained slit skin slides for amastigotes and also examined the wet film of culture for promastigotes.

**Examination for *M. leprae*:** At least two experts also looked for *M. leprae* in the Z-N stained slit skin slides.

***Definitions***

**Case definitions*:***  A suspected PKDL patient was a person from a kala-azar-endemic area with a past history of kala-azar and a skin lesion. A probable PKDL case was a patient with suspected PKDL who also had a positive rK39 test result. A confirmed PKDL case included those probably PKDL cases who also had the LD parasite identified by slit skin examination or culture, or had a PCR test positive for LD DNA. All the confirmed PKDL cases were referred to the Upazila Health Complex for treatment as per the national guideline for kala-azar elimination [17]. However, all analyses presented in this paper are based on clinically-defined PKDL with positive rK39 dipstick test, i.e. probable PKDL cases.

**Age-groups*:*** The study population was divided into three age-groups: <15 years; 15-43 years; and ≥44 years.

**Duration of onset of PKDL:** Duration was defined in months between the past treatment history of VL and the onset of skin lesion as reported by the patient.

**Grading of PKDL:** Skin lesions were graded into three major categories as described previously [1]. Briefly, grade I included scattered maculopapular or nodular rash in the face with or without some involvement in the upper chest or arm; grade II included maculopapular or nodular rash mostly of the face and extending to the chest, back, upper arms, and legs; and grade III included maculopapular or nodular rash covering most parts of the body, including hands and feet.

**Progression of skin lesions:** Based on case history given by the patients, the study physician categorized progression of skin lesions into rapid (conspicuous/obvious progression every month from onset), gradual (clear, steady progression from onset but at less detectable pace), and slow/stagnant (appearance, with little or no progression throughout early months). **Treatment categories:** Complete treatment was defined when a patient completed the full treatment course of 20 consecutive days per month for consecutive six months with sodium stibogluconate at a dose of 20 mg/kg/day and with a gap of 10 days between courses. Termination of treatment before its completion was defined as incomplete/partial treatment, and if a patient refused treatment with sodium stibogluconate, it was defined as refusal of treatment.

**Cure of PKDL:** Cure was defined as complete disappearance of skin lesion(s) after treatment, as reported by the patient and assessed by the trained Field Research Assistant.

*Calculation of sample-size and statistical analysis*

Assuming a PKDL prevalence of 0.04% (95% CI 0.03-0.05), the required sample-size needed to search for PKDL cases was 25,098. A total population of 29,226 in the study area was sufficient for our study. All data were computed using the EpiInfo software (version 3.2.2.) and were analyzed using the SPSS software (version 11.0) through descriptive and analytical methods. Comparison between proportions was done by chi-square with Fisher’s exact correction. Means were compared by ANOVA or Kruskal-Wallis where applicable. We used Kaplan-Meier survival curves to identify the median time for onset of PKDL after the completion of VL treatment. All p values were two-tailed, and a p value of ≤ 0.05 was considered significant.

***Ethical consideration***

The Ethical Review Committees of both ICDDR,B and Tropical Disease Research/WHO approved the study. Written informed consent was obtained from each study participant and from parents/legal guardian for all child participants. Written informed consent also was obtained from the head of each household when performing the census.

**Results**

***Prevalence of PKDL in study area***

The census performed by the Field Research Assistants revealed a total population of 29,226 people from 6,566 households: 11,298 people (38.7%) were aged <15 years, and the median family size within each household was 5 (IQR 4-6). The Field Research Assistants also listed a total of 235 individuals with a past history of VL and 21 suspected PKDL patients based on the information obtained during their visits to households at the beginning of the study. However, the TVVs referred 52 suspected PKDL patients, including the 21 cases, listed by the Field Research Assistants from the study area. No cases from the study area were self- reported but 13 patients were self-reported from villages outside the study area. No new cases of suspected PKDL from the study area were found in the following months after the initial survey by the TVVs.

Of the referred and self-reported suspected PKDL cases, 18 of 52 (34.6%) and 10 of 13 (76.9%) were respectively found to be probable PKDL cases. The referred and self-reported probable PKDL cases did not differ regarding age [mean years ±standard error (SE), 24.1±3.7 for referred cases, 18.5±3.3 for self-reported cases, p=0.33], sex (% of male patients, 55.6% for referred vs 40.0% self-reported, p=0.69), and duration of onset of PKDL (mean years±SE, 46.0±5.1 for referred vs 36.0±23.1 for self-reported cases, p=0.29). PKDL was confirmed in 50% of the patients from each group (9/18 for referred and 5/10 for self- reported suspected PKDL patients). The calculated prevalence of probable PKDL in the study area was 6.2 per 10,000 people, slightly more common among the age-group of 15-43 years and without any relation with gender [Table 1].

***Characteristics of PKDL patients***

We pooled all the 28 probable PKDL cases for further analysis. The median age of the patients was 21.5 years (IQR, 10.7–29.0), and the large majority (67.9%) was aged ≥15 years. All the patients had previously been treated for VL with sodium stibogluconate for 28 consecutive days, except one (3.6%) who had interruption in treatment. The median duration of onset of PKDL was 36 months (IQR, 24-48) (Fig. 1). The duration of onset of PKDL was not related to gender (38.57±5.9, n=14 for females vs 46.71±5.9, n=14 for males, p=0.34) but the younger group developed PKDL significantly earlier compared to the older age-group (26.67±3.8 months, n=9, vs 50.21±5.1 months, n=19, p=0.006).

Hypopigmented macules were the most common skin lesions and most frequently appeared first on the face (Table 2). In most (94.4%) cases, the skin lesions progressed gradually and were associated with itching in 25% of the cases. Eight of the 28 (29%) cases, skin lesions were classified as grade I and the remaining 20 (71%) as grade II at the time of presentation to the study clinic. The severity of the disease, as estimated by grading, showed no association with sex: 11 of the 14 females (78.6%) and 9 of the 14 males (64.3%) had grade II PKDL, p=0.34. Similarly, the severity of the disease had no association with age-group (78% vs 68% grade II respectively in the younger and older age-groups, p=0.48) nor the duration of onset of PKDL (r=0.03, p=0.9).

***Laboratory findings***

All the patients were negative for *M. leprae* (Table 3). All the 28 cultures were negative for Leishmania parasites. Slit skin microscopy found LD bodies only in one of the 28 patients. Of the 28 patients 12 (42.9%) were positive by slit skin scraping specimen PCR and 14 (50%) by peripheral blood buffy coat PCR. The PCR method was positive in 64% of the patients, defined by any PCR test-positive (Table 3). The buffy coat PCR results were positively associated with the severity of the disease: 65% (13/20) of grade II PKDL patients were positive for peripheral buffy coat PCR compared to 12.5% (1/8) of the PKDL patients with grade I disease (p=0.012). However, skin specimen PCR results did not show a similar relationship with PKDL grades: 45% (9/20) and 37% (3/8) respectively with grade II PKDL and grade I PKDL were positive by skin specimen PCR (p= 0.72). The female patients had significantly more peripheral buffy coat PCR-positive results compared to the male patients[ 10/14 (71%) for female vs. 4/14 (29%) for male patients, p= 0.023]. The female patients were also more likely to have a positive skin specimen by PCR (50%, 7/14) compared to the male patients (36%, 5/14) but the difference was not statistically significant (p=0.45).

***Treatment and follow-up***

Eighteen patients positive for the evidence of the LD parasite (1 by both LD body and LD DNA and 17 by LD DNA only) by any laboratory method were referred to the subdistrict hospital for treatment. Of these patients, 7 completed treatment, 6 partially completed treatment, and 5 refused treatment. The common reasons for incomplete treatment or not to be treated were concern about loss of daily wages, loss of school days, and insolvency (Table 4). All the patients with complete treatment, half of those with incomplete treatment, and none who refused treatment were cured at follow-up. Compared to those with complete treatment, the risk of not being cured with incomplete treatment or treatment refusal was three times higher (relative risk 3.33, 95% CI 1.29–8.59, p=0.004). Ten of the 28 patients were not referred for treatment due to lack of evidence of the LD parasite in their skin or blood. At follow-up after one year, 8 of 10 patients had persistent skin lesions; skin lesions had deteriorated in 1 patient, and 1 was spontaneously cured.

**Discussion**

The major findings of the present study were: the TVVs were useful for the active detection of PKDL case; the prevalence of PKDL was high in the study area; PCR was useful for the confirmation of PKDL diagnosis; and low treatment compliance and current treatment-seeking behavior of PKDL patients present a new challenge for the national kala-azar elimination programme in Bangladesh.

The Government of Bangladesh is committed to eliminate VL by 2015, and active detection of VL and PKDL cases is one of the main pillars of this elimination programme [8, 17]. The active detection of VL and PKDL cases by the existing health facilities in rural Bangladesh may not be possible due to lack of human resources. We found that the TVVs were useful for finding suspected PKDL cases. Thus, the TVVs should be used by the NKEP for an annual active case search. We feel that it is sufficient to conduct an annual search, particularly for PKDL, since no new cases of suspected PKDL were found in our study after the initial active case search by the TVVs.

The proportion of PKDL cases among the self-reported suspects was higher (76.9%) compared to the referred PKDL cases (34.6%). The majority of the self-reported cases were directed to our study clinic from nearby public union health posts that lacked facilities for rK39 testing. This suggests that many people are concerned about signs and symptoms of possible PKDL but remain undiagnosed due to lack of diagnostic facilities, particularly rK39-based rapid tests, in the union health posts. The NKEP should thus, strengthen the existing public union health posts in the VL-endemic areas by equipping them with tools to diagnose PKDL.

Clinical manifestations of the PKDL cases did not differ from those reported in the literature [1-6,18]. We found that the median duration of onset of PKDL was slightly longer compared to that reported recently [4]. This difference might be explained by the differences in the study design, and recall bias in our study population also might contribute to this difference. Itching was reported in 25% of the cases. This symptom among the PKDL patients was also reported by others, and inflammatory reactions in the dermis observed by Ismail *et al* might explain this symptom [19].

The prevalence of PKDL in our study was 6.2 per 10,000 people which was lower than that found by a recent study in another VL-endemic area of Bangladesh [3]. The limitation of our study was that the TVVs looked for the suspected PKDL cases based on the past history of VL and skin rash. Since PKDL may develop in individuals without a past history of VL, it was possible that the TVVs could not track all suspected PKDL cases. However, we believe that this risk was minimal because the percentage of PKDL without past history of VL was low (about 10% of all PKDL cases) in Bangladesh [4]. Additionally, no self-reported PKDL case from Kanthal union was presented to the study clinic or the Upazila Health Complex in the months following the study.

Slit skin examination and culture methods were not very much useful for the diagnosis of PKDL since the LD parasite was found in only one by slit skin examination, and all the 28 cases were negative by culture method. Similar results were reported by others [18]. PCR of skin specimens and peripheral blood buffy coat was more useful for the detection of LD compared to slit skin examination and culture. PCR helped confirm the diagnosis of PKDL in additional 17 patients who were negative for LD by conventional methods. Seven of the 17 patients who received complete treatment were cured. Without this useful diagnostic tool, these 7 patients would not be considered PKDL patients because they were negative by SSE and culture and would continue to serve as a reservoir of anthroponotic VL, thus threatening the community’s health. In Bangladesh, many tertiary hospitals are now equipped with PCR technology. Thus, the NKEP should consider the use of PCR for the diagnosis of PKDL. However, the feasibility and cost-effectiveness of such a diagnostic algorithm may be a concern for the NKEP and needs to be explored first.

We observed a significantly-positive relationship between PKDL grade and PCR positivity, particularly with peripheral blood buffy coat PCR. The circulation of the LD parasites in peripheral blood increases with the severity of disease and indirectly indicated that the origin of LD DNA in blood was skin. So far, this is the first documentation of such a relationship between PKDL grade and PCR method. It also supports an earlier experiment that patients with PKDL may serve as a reservoir for the transmission of the LD parasite which circulates in their blood [20]. Although the severity of PKDL was equally distributed among the males and females, the positive PCR results were more common in the females, especially for peripheral buffy coat PCR. We have no explanation for this phenomenon.

Ten of the 28 probable PKDL cases were not referred for treatment because we failed to demonstrate LD or LD DNA among them. At follow-up, only one had improvement in his skin rashes, which occurred spontaneously. Our current diagnostic capabilities limited our ability to determine whether these 10 cases were false-positive probable PKDL cases or if they were false-negative confirmed PKDL cases. The former explanation is less likely to be true because PKDL is typically not self-limited in the Indian sub-continent and less likely to be cured without treatment [1-6]. All except one patient had persistent skin lesions after a one year in this study. Thus, the development of more sensitive tests for the diagnosis of PKDL is urgently needed and should be encouraged by national and international researchers and funding agencies.

The poor treatment-seeking behavior of and treatment compliance by patients with PKDL patients present a challenge for the NKEP. Only seven of the 18 cases (38.9%) completed treatment after referral to the hospital. The reason behind not being treated and partially treated was respectively feeling otherwise healthy and concern about loss of daily wage or loss of school day. Intensive motivation and some financial support from the NKEP may improve this situation.

In conclusion, the prevalence of PKDL is high in a VL-endemic area of Bangladesh. The use of TVVs feasible to actively detect PKDL suspects, and in conjunction with PCR techniques, this holds promise as an effective strategy for the NKEP to help meet goals for the elimination of VL, if the ways for improving treatment-seeking behavior and treatment compliance are found.

**Acknowledgements**

Special Programme for Research and Training in Tropical Diseases (TDR), World Health Organization, Geneva, Switzerland funded the study (Project ID A60443B).The study has been conducted with the support of ICDDR,B, and ICDDR,B acknowledges with gratitude the commitment of UNICEF/UNDP/World Bank/WHO to the Centre’s research efforts. We are also grateful to all the study participants. We are thankful to Mr. Md. Golam Rabbani, Chairman, Kanthal union, Trishal, Mymensingh for his kind cooperation in the selection of community volunteers and for accommodating the research team in Kanthal. We acknowledge with gratitude the contributions of Md. Didarul Islam, Arif Rabbani, Bayeazid Bostami, Mostafijur Rahman, Md. Ershad Ali, Md. Sohrab Uddin, Lalmohan, Md. Motiur Rahman, and Md. Ruhul Amin who contributed to this study as village volunteers. We are thankful to Mr. Samsul Islam Khan, Head, Publication, ICDDR,B for language editing of the manuscript.

**References**

1. Zijlstra EE, Musa AM, Khalil EAG, El Hassan IM, El-Hassan AM (2003). Post kala-azar dermal leishmaniasis. Lancet Infectious Diseases 3: 87-97.
2. Zijlstra EE, El-Hassa AM (2001), Leishmaniasis in Sudan. Post kala-azar dermal leishmaniasis. Trans Roy Soc Trop Med Hyg 95: supplement 1, S1/64-S1/76.
3. Thakur CP, Kumar A, Mitra G, Thakur S, Sinha PK, Das P, Bhattacharya SK, Sinha A (2008). Impact of amphotericin-B in the treatment of kala-azar on the incidence of PKDL in Bihar, India. Indian J Med Res 128: 38-44.
4. Rahman KM, Islam S, Rahman MW, Kenah E, Galive CM, Zahid MM, Maguire J, Rahman M, Haque R, Luby SP, Bern C (2010). Increasing incidence of post-kala-azar dermal leishmaniasis in a population-based study in Bangladesh.Clin Infect Dis 50: 73-76.
5. Garg VK, Agrawal S, Rani S, Joshi A, Agrawalla A, Das ML, Koirala S (2001). Post-kala-azar dermal leishmaniasis in Nepal. International Journal of Dermatology 40: 179-184.
6. Kordofani YM, Nour YT, El-Hassan AM, Shalayel MH (2001). Post kala-azar dermal leishmaniasis in Sudan. East Mediter Health J 7: 1061-1064.
7. Addy M, Nandy A. Ten years of kala-zar in West Bengal, Part I (1992). Did post-kala-azar dermal leishmaniasis initiate the outbreak in 24-Parganas? Bulletin of the World Health Organization 70: 341-346.
8. World Health Organization. Regional Technical Advisory Group on Kala-azar Elimination. Report of the first meeting, Manesar, Haryana, 20-23 December 2004 (2005). Regional Office for South-East Asia, New Delhi, October **2005**.
9. Mondal D, Singh SP, Kumar N, Joshi A, Sundar S, Das P, Siddhivinayak H, Kroeger A, Boelaert M. Visceral leishmaniasis elimination program in India, Bangladesh, and Nepal: Reshaping the case finding/case management strategy (2009). PLoS Negl Trop Dis 3: e355.
10. Brahmachari UN (1922). A new form of cutaneous leishmaniasis dermal leishmanoid. Indian Med Gaz: 57: 125.
11. Salotra P, Sreenivas G, Beena KR, Mukherjee A, Ramesh V. Parasite detection in patients with post kala-azar dermal leishmaniasis in India: a comparison between molecular and immunological methods (2003). J Clin Pathol 56: 840-843.
12. Cruz I, Canavate C, Rubio JM, Morales MA., Chicharro C, Laguna F, Jimenez-Mejias M, Sierra G, Videla S, Alvar J (2002). A nested polymerase chain reaction (Ln-PCR) for diagnosing and monitoring Leishmania infantum in patients co-infected with human immunodeficiency virus. Trans. Roy. Soc. Trop. Med. Hyg 96: 185-189.
13. Salam MA, Mondal D, Kabir M, Haque R. PCR for diagnosis and assessment of cure in kala-azar patients in Bangladesh (2010). Acta Tropica 113: 52-55.
14. Chowdhury AHK, Rahman J, Khan H, Tahir M (1988). Post kala-azar dermal leishmaniasis (PKDL) study of 8 cases. Bangladesh Medical Journal 17: 27-33.
15. El-Masum MA, Evans DA. Characterization of *Leishmania* isolated from patients with kala-azar and post kala-azar dermal leishmaniasis in Bangladesh (1995). Trans Roy Soc Trop Med Hyg 89: 331-332.
16. Salotra P, Sreenivas G, Pogue GP, Lee N, Nakhasi HL, Ramesh V, Negi NS. Development of species-specific PCR assay for detection of Leishmania donovani in clinical samples from patients with kala-azar and post-kala-azar dermal leishmaniasis (2001). J Clin Microbiol 39: 849-854.
17. Rahman, R., Bangali, M., Kabir, H., Naher, F.B., Mahboob, S. (2008). Kala-azar situation in Bangladesh. In: Hossain, M. (Ed.), National Guideline and Training Module for Kala-azar Elimination in Bangladesh. First edition, January 2008. CDC, DGHS, Ministry of Health and Family Welfare, Government of the Peoples Republic of Bangladesh. pp. 11-12.
18. Salotra P, Singh R. Challenges in the diagnosis of post kala-azar dermal leishmaniasis (2006). Indian J Med Res 123: 295-310.
19. Ismail A, Khalil EAG, Musa AM, El Hassan IM, Ibrahim ME, Theander TG, El Hassan AM (2006). The pathogenesis of post kala-azar dermal leishmaniasis from the field to the molecule: Does ultraviolet light (UVB) radiation play a role? Medical Hypothesis 66: 993-999.
20. Monila R, Canavate C, Cercenado E, Laguna F, Velez RL, Alvar J (1994). Indirect xenodiagnosis of visceral leishmaniasis in 10 HIV-infected patients using colonized *Phlebotomaus perniciosus.* AIDS 8: 277-279.

**Title and Figure legend**

Fig. 1 Time for developing PKDL after completion of treatment for visceral leishmaniasis

Table 1 Distribution and prevalence of probable PKDL patients by sex and age group in Kanthal

|  | Total number of people in the study area | Number of probable PKDL Patients | Prevalence of probable PKDL per 10,000 people |
| --- | --- | --- | --- |
| Sex |  |  |  |
| Male | 14999 | 10 | 6.7 ^a^ |
| Female | 14227 | 08 | 5.6 |
| Age group |  |  |  |
| < 15 years old | 10569 | 05 | 4.7 |
| 15-43 years old | 13535 | 11 | 8.1 ^b, c^ |
| ≥ 44 years old | 5122 | 02 | 3.9 |
| Total | 29226 | 18 | 6.2 |

^a^ P = 0.72 compare to Female group; ^b^ P= 0.31 vs. < 15 years old and ^c^ P= 0.53 vs. ≥ 44 years old

Table 2 Clinical information of probable PKDL patients, n=28

| Indicators | % (n) |
| --- | --- |
| First involvement of rash |  |
| Face | 60.7 (17) |
| Arms | 28.6 (8) |
| Other part of the body | 10.7 (3) |
| Rash progression |  |
| Gradual | 96.4 (27) |
| Slow/stagnant | 3.6 (1) |
| Type of rashes |  |
| hypopigmented macules/patches/confluent plaques | 100.0 (28) |
| hypopigmented papules | 10.7 (3) |
| nodule | 3.6 (1) |
|  |  |

Table 3 Laboratory findings in 28 probable PKDL patients

| Laboratory Method | Positive % (n/28) |
| --- | --- |
| Microscopy for M. Leprae | 0 (0) |
| Culture for Leishmania promastigote | 0 (0) |
| Microscopy of slit skin scraping specimens | 3.6 (1) |
| PCR |  |
| Slit skin scraping specimens | 42.9 (12) |
| Peripheral buffy coat | 50.0 (14) |
| Any positive | 64.3 (18) |

Table 4. Laboratory diagnosis, referral and treatment compliance of probable PKDL patients

| Patient | Age  ( years) | Sex | PKDL Grade | Slit skin examination | Culture  of skin  specimens  for LD | *PCR Result | Referred  for treatment | Treatment status (number of injections) | Reason of partial treatment or not treated | Status after one year |
| --- | --- | --- | --- | --- | --- | --- | --- | --- | --- | --- |
| T-085 | 26.00 | Male | 2 | Negative | Negative | 2 | Yes | Partial (18) | Loss of wages | Not cured |
| T-013 | 30.00 | Female | 2 | Negative | Negative | 3 | Yes | Complete (120) | - | Cured |
| T-073 | 7.00 | Female | 2 | Negative | Negative | 3 | Yes | Complete (120) | - | Cured |
| T-034 | 34.33 | Female | 1 | Negative | Negative | 2 | Yes | Complete (120) | - | Cured |
| T-035 | 25.00 | Female | 3 | Negative | Negative | 3 | Yes | Complete (120) | - | Cured |
| T-015 | 7.00 | Female | 2 | Negative | Negative | 1 | Yes | Partial (54) | Insolvency | Not cured |
| T-019 | 26.00 | Male | 2 | Negative | Negative | 3 | Yes | Complete (120) | - | Cured |
| T-028 | 45.00 | Male | 2 | Negative | Negative | 3 | Yes | Partial (59) | Insolvency | Not cured |
| T-084 | 13.00 | Female | 1 | Negative | Negative | 3 | Yes | Complete (120) | - | Cured |
| T-040 | 22.00 | Male | 1 | Negative | Negative | 2 | Yes | Partial (62) | Insolvency | Cured |
| T-041 | 17.00 | Female | 2 | Negative | Negative | 2 | Yes | Partial (40) | Loss of school days | Cured |
| T-042 | 10.00 | Female | 2 | Negative | Negative | 1 | Yes | Partial (40) | Loss of school days | Cured |
| T-057 | 7.00 | Female | 2 | Negative | Negative | 1 | Yes | Complete (120) | - | Cured |
| T-022 | 70.00 | Male | 2 | Negative | Negative | 0 | No | Not referred | - | Cured |
| T-072 | 25.00 | Male | 1 | Negative | Negative | 0 | No | Not referred | - | Not cured |
| T-074 | 2.25 | Male | 2 | Negative | Negative | 0 | No | Not referred | - | Not cured |
| T-087 | 20.00 | Male | 2 | Negative | Negative | 0 | No | Not referred | - | Not cured |
| T-088 | 30.00 | Female | 1 | Negative | Negative | 0 | No | Not referred | - | Not cured |
| T-120 | 13.00 | Male | 1 | Negative | Negative | 0 | No | Not referred | - | Not cured |
| T-121 | 10.00 | Male | 2 | Negative | Negative | 0 | No | Not referred | - | Not cured |
| T-123 | 25.00 | Female | 2 | Negative | Negative | 0 | No | Not referred | - | Rash increased |
| T-124 | 2.50 | Female | 2 | Negative | Negative | 1 | Yes | Not taken | Does not feel sick | Not cured |
| T-126 | 19.00 | Male | 1 | Negative | Negative | 0 | No | Not referred | - | Not cured |
| T-141 | 21.00 | Male | 1 | Negative | Negative | 0 | No | Not referred | - | Not cured |
| T-029 | 25.00 | Male | 2 | Negative | Negative | 1 | Yes | Not taken | Loss of wages | Not cured |
| T-063 | 30.00 | Female | 2 | Negative | Negative | 1 | Yes | Not taken | Does not feel sick | Not cured |
| T-086 | 38.00 | Female | 2 | Negative | Negative | 3 | Yes | Not taken | Does not feel sick | Not cured |
| T-110 | 20.00 | Male | 2 | Positive | Negative | 3 | Yes | Not taken | Loss of wages | Not cured |

*0= Negative both by skin specimen PCR and peripheral blood buffy coat PCR, 1= positive by only peripheral buffy coat PCR, 2= positive only by skin specimen PCR, 3= Positive by both PCR
